# Multiconfigurational Pair-Density Functional Theory Is More Complex than You May Think

**DOI:** 10.1021/acs.jpca.3c05663

**Published:** 2023-10-27

**Authors:** Gabriel L. S. Rodrigues, Mikael Scott, Mickael G. Delcey

**Affiliations:** † Division of Theoretical Chemistry and Biology, School of Engineering Sciences in Chemistry, Biotechnology and Health, KTH Royal Institute of Technology, Stockholm SE-100 44, Sweden; ‡ Division of Theoretical Chemistry, Department of Chemistry, Lund University, Lund SE-221 00, Sweden

## Abstract

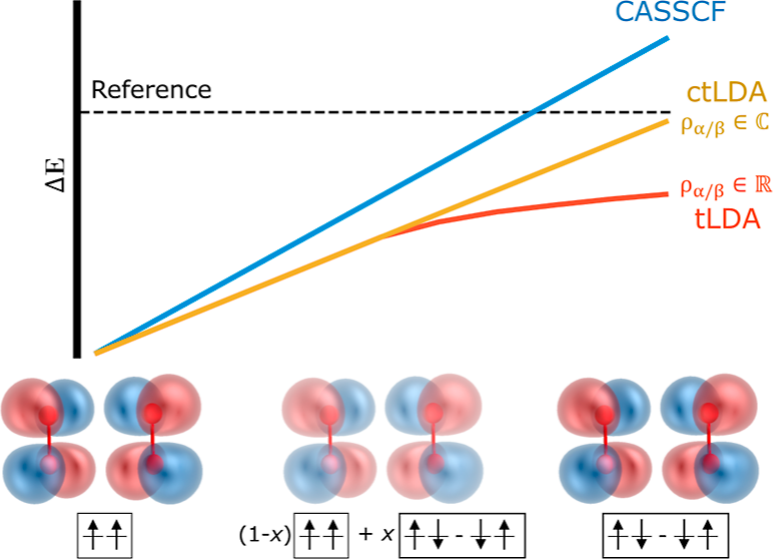

Multiconfigurational pair-density functional theory (MC-PDFT) is a promising way to describe both strong and dynamic correlations in an inexpensive way. The functionals in MC-PDFT are often “translated” from standard spin density functionals. However, these translated functionals can in principle lead to “translated spin densities” with a nonzero imaginary component. Current developments so far neglect this imaginary part by simply setting it to zero. In this work, we show how this imaginary component is actually needed to reproduce the correct physical behavior in a range of cases, especially low-spin open shells. We showcase the resulting formalism on both local density approximation and generalized gradient approximation functionals and illustrate the numerical behavior by benchmarking a number of singlet–triplet splittings (ST gaps) of organic diradicals and low-lying excited states of some common organic molecules. The results demonstrate that this scheme improves existing translated functionals and gives more accurate results, even with minimal active spaces.

## 1 Introduction

Inherently multideterminantal systems are often called strongly correlated and are usually poorly described by the standard Kohn–Sham density functional theory (KS-DFT) due to its single configurational nature. At the same time, while multiconfigurational methods such as the multiconfigurational self-consistent field (MCSCF)^1^ and in particular the complete active space self-consistent field (CASSCF)^2^ are efficient in capturing the strong correlation of a system, they often fail to obtain quantitative energies or properties if the dynamic correlation is not properly treated, which is usually done in a post-SCF way by relatively expensive perturbative methods such as CASPT2^3^ or NEVPT2.^4^ For this reason, it is a very tempting idea to develop a multiconfigurational DFT method, aiming to combine the rigorous treatment of static/strong correlation of MCSCF with the cost-efficiency of DFT to treat dynamic correlation.

This combination of MCSCF with DFT has been pursued for several years through the development of various methods.^5−17^ Among them, the multiconfigurational pair-density functional theory (MC-PDFT) approach is one of the most popular, largely due to the implementation by Li Manni and co-workers.^18^ There, they suggested a two-step process: first optimize the MCSCF wave function and then use the obtained one- and two-particle densities to compute the PDFT energy. The PDFT energy itself can be seen as an extension of the KS-DFT energy with a MCSCF wave function and a functional written in terms of density and on-top pair-density (*E*_ot_[ρ, Π])
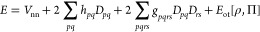
1where *p*, *q*, *r*, and *s* are the indices for general molecular orbitals, *V*_nn_ is the sum of the nuclear repulsion, *h*_*pq*_ and *g*_*pqrs*_ are the one- and two-electron integrals, respectively, and *D*_*pq*_ is the one-electron density matrix.

One of the most well-known issues with multiconfigurational DFT is double counting due to the fact that both MCSCF and DFT account for dynamical correlation within the active space. However, MC-PDFT avoids this since only the PDFT energy is computed, with the MCSCF energy simply dropped. The resulting method has a computational cost essentially identical to MCSCF, but it has been found to produce results of CASPT2 quality. In its original article, MC-PDFT showed promising results in benchmarks^18^ of dissociation and excitation energies of small molecules and atoms, reducing the error at the CASSCF level by an average of almost half while being 20% more accurate than regular KS-DFT, and indeed obtaining similar accuracy as the much more expensive CASPT2 method. After that, other studies from the same group^19^ discovered that MC-PDFT is even better than CASPT2 for calculating atomization energies of main group elements and transition metals, while having performance between CASPT2 and CASSCF for barrier heights and reaction energies.^20^ Singlet–triplet splittings of organic diradicals were also tested^21^ where MC-PDFT showed an accuracy comparable of CASPT2. However, these tests also revealed a significant active space dependence of the MC-PDFT results, which is highly undesirable and also surprising since one would hope for MC-PDFT to be at least as good as KS-DFT for a minimal active space.

While in principle new functionals would need to be designed for PDFT, it is common practice to simply use a standard KS-DFT code with effective spin densities computed from their relation with density and on-top pair-density for a single determinant
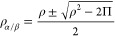
2

This guarantees that the PDFT functional gives the same result as the corresponding KS-DFT functional for a single determinant. However, for a multiconfigurational wave function, the quantity under the radical in eq 2 is not guaranteed to be positive. Yet, to our knowledge, all previous implementations simply neglected these cases, without a more in-depth discussion on how common or relevant they are.

In this article, we look at the physical meaning of having “complex effective spin densities” and show how this is actually a fairly common situation in many relevant applications. We then illustrate a general proper functional translation and show how this yields improved accuracy for these cases and, in particular, results in a reduced active space dependence.

## 2 Theory

### 2.1 Pair-Density Functional Theory

The idea of MC-PDFT actually originates from the suggestion in 1991 by Moscardó and San-Fabián,^7^ later extended by Becke, Savin, and Stoll,^22^ to introduce two-body effects into the density functional by taking into account the on-top pair-density Π. They showed how this would, among others, improve the description of potential energy curves of pure spin diatomic molecules and ensure the correct degeneracy of the three triplet components.

For a single determinant, ρ and Π share a simple relation with the usual spin densities
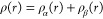
3and
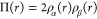
4

With these relations, it is possible to “translate” existing SDFT functionals to use the total and on-top pair-densities while still ensuring that they yield the same results for a single determinant case. The simplest way to achieve this is to invert eqs 3 and 4 to compute the “effective” spin densities from the total and on-top pair-densities. Since the resulting equation is a second-order polynomial, we obtain a pair of solutions which correspond to the α and β densities as illustrated in eq 2. By convention, we can choose that the α density corresponds to the “+” term and β to the “–” term.

Interestingly, while by construction such a translation provides the standard SDFT results for a single determinant, it gives different and more physically accurate results for several determinants. In particular, this formulation enables the *m*_*s*_ = 0 component of the triplet to be degenerate with the other two, and it also immediately enables a qualitatively correct description of bond-breaking and other static correlation problems.

### 2.2 Analytic Continuation to Complex Spin Densities

The presence of a square root in eq 2 demands a more careful consideration. Let us denote the quantity under the radical as
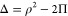
5

Physically, the on-top pair-density is always bounded as 0 ≤ Π(*r*) ≤ ρ^2^(*r*), which means −ρ^2^(*r*) ≤ Δ(*r*) ≤ ρ^2^(*r*).

We will come back to the meaning of the different ranges in the next subsection. For now, it suffices to say that there are cases where the translated spin densities from this formula become complex. In order to properly treat these cases, one can go back to the work of Becke et al.,^22^ where they noticed that by simply allowing the spin densities to have an imaginary component, the yielded energies remain real since a correct DFT functional should be an even power of the spin polarization. In particular, they illustrated how the SLDA exchange term would look when extended to the complex algebra formalism.

**Figure 1:**
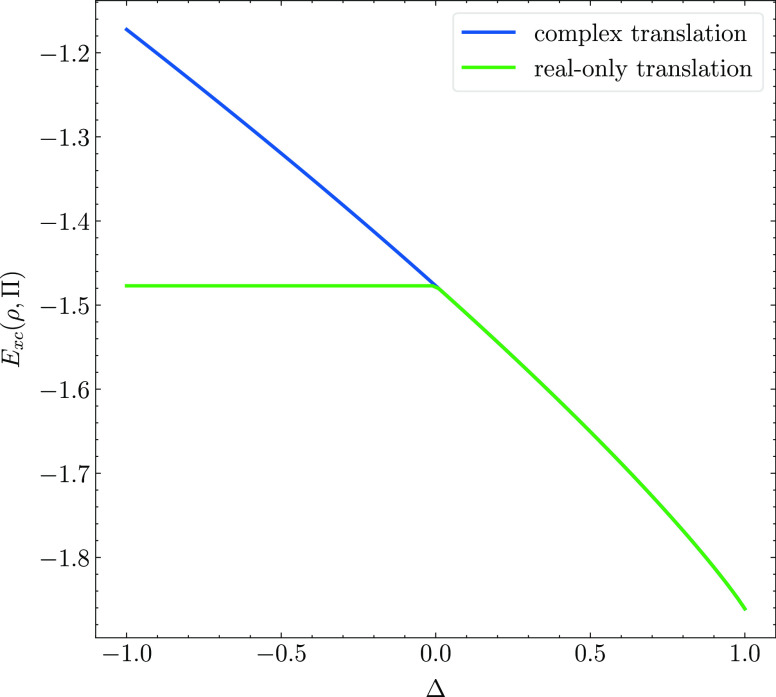
Slater exchange energy for a range of Δ (eq 5) values when ρ = 1.0 au and Π linearly varies from 0 to 1. For the complex translation, the Slater exchange energy is calculated according to eq S5 or S6 from the Supporting Information, if Δ ≥ 0 or Δ < 0, respectively. In the case of real-only translation, eq S5 is always used, but Δ is set to 0 when ρ^2^ – 2Π < 0.

In the Supporting Information, we show how this is done, in particular, for the LSDA and PBE functionals. We can check the outcome of this approach with a plot of the Slater exchange energy against the entire range of possible Δ values (as defined in eq 5), for a constant density ρ = 1.0 au, as shown in Figure 1. As can be seen, for a range of possible Δ values from −1 to 1, the functional is perfectly smooth, contrary to the real-only translation, where we have a derivative discontinuity at Δ = 0. In order to allow derivatives for GGA functionals, the so-called “fully translated functionals” have been proposed, but while these functionals remove the kink in an ad hoc manner, they do not change the broad behavior.^23^ To avoid divergence of the density gradients for GGAs, we use the same translation scheme as proposed first by Li Manni et al., which neglects the contribution from the gradient of the on-top pair-density.

### 2.3 Physical Meaning of Complex Translated Densities

Let us go back to the physical meaning of these situations.

For a closed-shell single determinant, eq 4 simplifies to  and Δ = 0. Therefore, Δ can be understood as the change in the on-top pair-density compared to a closed-shell Hartree–Fock case. In most situations, Δ is positive, reflecting the fact that adding determinants add correlation, which tends to decrease the probability of having two electrons in the same position and thus decrease Π(*r*).

However, in some cases, it can indeed become negative. Since the on-top pair-density is a measure of the exchange–correlation hole, these cases can be understood as an overestimation of the exchange hole in Hartree–Fock. Thus, improving the wave function increases the probability of finding two electrons at the same point in space.

This difficulty was already noted in the original 1995 article, and the authors even presented a simple example where this would happen, namely, a spin-adapted open-shell singlet.^22^ We can see this in the triplet (*E*_T_), broken symmetry (*E*_BS_), and open-shell singlet (*E*_S_) energies for a two-electron, two-orbital system using a bar to denote spin-down electrons
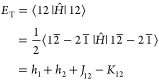
6
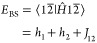
7
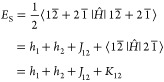
8where *h*_*p*_ is one-electron energy for orbital *p* and *J*_*pq*_ = (*pp*|*qq*) and *K*_*pq*_ = (*pq*|*qp*), respectively, are the Coulomb and exchange integrals. Clearly, going from the broken symmetry single determinant to the true singlet, we have reduced the correlation in a mathematical sense and increased the overall energy. Due to its role and form, it is reasonable to actually consider this term a part of the exchange energy, and in this case, we can say that the exchange energy is reduced (note that the exchange also includes the self-interaction canceling terms not shown in these equations, meant to remove the double counting of *J*_11_ and *J*_22_ in the Coulomb energy).

Thus, we can see that the analytical continuation behavior shown in Figure 1 is physically sound, representing a decrease in the exchange energy for negative Δ.

Note that while we only showed the case for a two-electron, two-orbital open-shell singlet, any low-spin open-shell (i.e., any open-shell where the total multiplicity is less than the total number of open-shell electrons plus one) will display such a behavior. Such configurations are very common and are present in many metal complexes but also in some organic diradicals or even the most excited states. Because of this, neglecting the imaginary part can have dramatic consequences for the calculated energies and derived properties in a wide range of systems.

## 3 Calculation Details

All the translated SDFT functionals have been renamed by just including a lowercase t before their original name. Additionally, a lowercase c was added when using the complex translation. Thus, we have tLDA, tPBE, and tBLYP as the standard translated PDFT functionals and ctLDA, ctPBE, and ctBLYP for our complex translation. These translations will be called “real-only” and “complex” translations. In the real-only translation, whenever Δ is negative, we set Π to zero, similarly to the translation in the original MC-PDFT paper.^18^

For both singlet–triplet gaps and excitation energies, we show the results with both a minimal active space consisting of two electrons and two orbitals (denoted minimal) and, for relevant molecules, another active space where all π orbitals were included as well as possible high-lying *n* orbitals necessary for the excitations (denoted as π). Dunning’s cc-pVDZ^24^ was used for the singlet–triplet splittings, while the augmented aug-cc-pVDZ^25^ basis sets were used for the excitation energies.

The first cases of open-shell singlets in this work are diradical molecules, where as mentioned before, the singlet–triplet splitting can be highly affected by the neglected correlation of a real-only functional translation. We used a set of molecules from the Stoneburner^26^ and Bao^27^ benchmarks. The sizes of the π active spaces for the relevant molecules are shown in Table 1.

**Table 1:** Active Space (π) of *n* Electrons and *m* Orbitals for Molecules where Singlet–Triplet Splittings Were Computed with Extended Active Spaces

molecule	(*n*,*m*)
O_2_	(6,4)
C_5_H_5_^+^	(4,5)
C_4_H_2_-(13)-2CH_2_	(6,6)
C_4_H_4_	(4,4)
C_4_H_2_–NH_2_	(6,5)
C_4_H_2_–CHO	(6,5)
C_4_H_2_–NH_2_–CHO	(8,7)

As a second category of example, we use perhaps the most commonly encountered open-shell singlet systems, namely, excited states produced by singlet–singlet transitions. Here, we have evaluated *S*_0_ → *S*_1_ excitations for the molecules that are present in the benchmark sets of both Hoyer and co-workers^20^ and Ghosh and co-workers.^17^. The molecules are acetone, formaldehyde, pyrazine, pyridazine, pyridine, pyrimidine, *s*-tetrazine, ethene, butadiene, benzene, naphthalene, furan, and hexatriene.

The excitation energies were computed by the difference between the energetically lowest two states of a state-averaged complete active space (SA-CASSCF) calculation, where for the π active space, the number of states was chosen as low as possible to stabilize the MCSCF. MC-PDFT energies were obtained by using the densities for each state in SA-CASSCF. The active space summary for each molecule can be seen in Table 2.

**Table 2:** Active Space (π) of *n* Electrons and *m* Orbitals for Each Molecule where *S*_0_ → *S*_1_ Excited States Were Computed

molecule	states	(*n*,*m*)	main transition
acetone	2	(4,3)	*n* → π*
formaldehyde	2	(4,3)	*n* → π*
pyrazine	5	(10,8)	*n* → π*
pyridazine	5	(10,8)	*n* → π*
pyridine	3	(8,7)	*n* → π*
pyrimidine	5	(10,8)	*n* → π*
*s*-tetrazine	6	(14,10)	*n* → π*
ethene	2	(2,2)	π → π*
butadiene	2	(4,4)	π → π*
benzene	2	(6,6)	π → π*
naphthalene	2	(10,10)	π → π*
furan	2	(6,5)	π → π*
hexatriene	2	(6,6)	π → π*

All calculations were performed in the MultiPsi program.^28^

## 4 Results and Discussion

### 4.1 Singlet–Triplet Splittings

**Figure 2:**
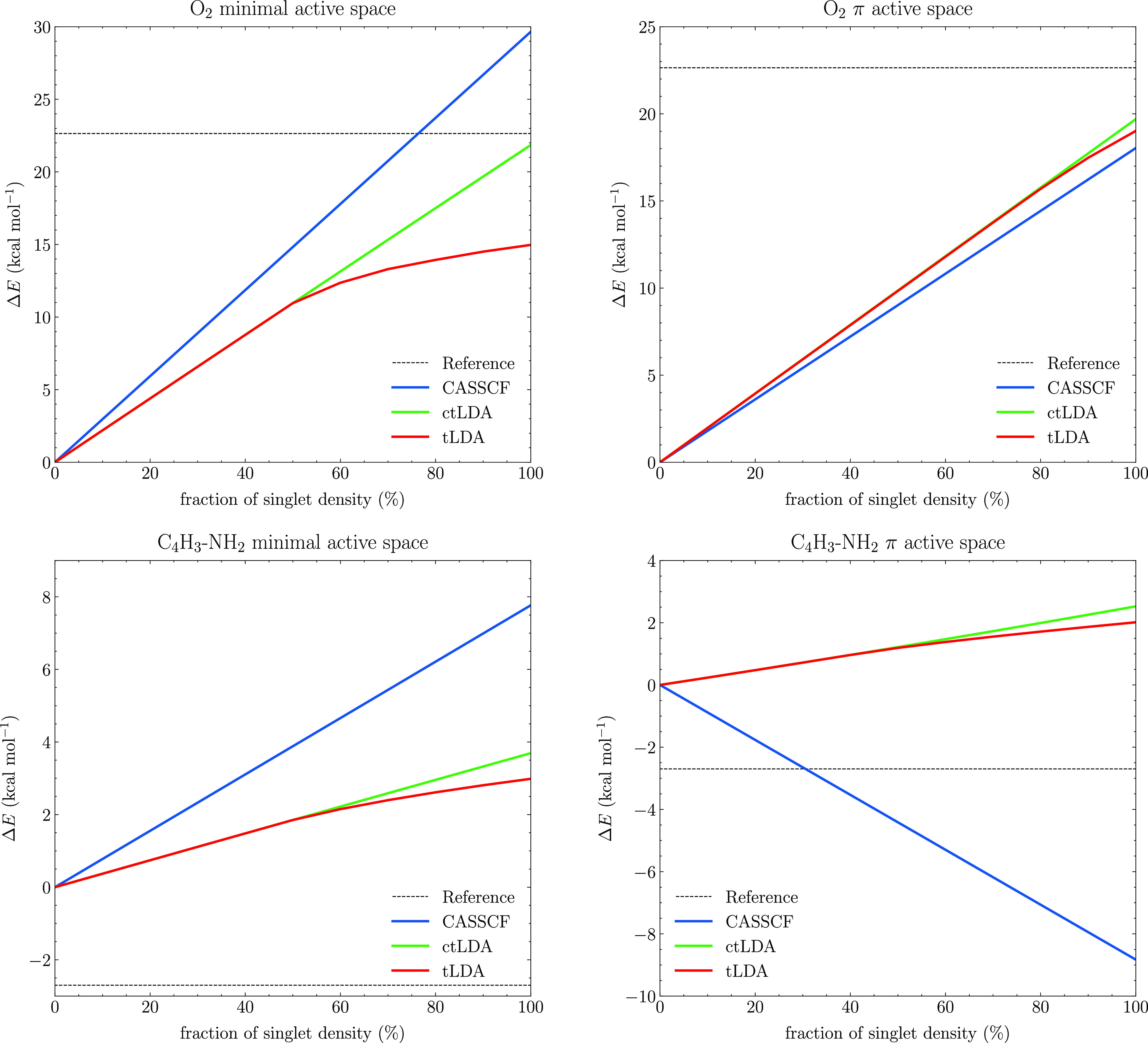
Triplet gap analysis for O_2_ and C_4_H_3_–NH_2_ molecules. The calculations were performed using the cc-pVDZ basis set using CASSCF, MC-tLDA, and MC-ctLDA. The gaps are analyzed by calculating the energy for a set of densities that are linearly interpolated between the triplet and the singlet.

Before analysis of the set of singlet–triplet splitting, it is interesting to look at the effect of the translation on a more artificial situation to gain some insight. In Figure 2, we plot the CASSCF and MC-PDFT energies (with the tLDA functional) for O_2_ and C_4_H_3_–NH_2_ on an interpolation path between the triplet and singlet state. We did this by simply linearly interpolating the one- and two-particle density matrices between the triplet and singlet states (*D*_int_ = *cD*_T_ + (1 – *c*)*D*_S_ with *c* going from 0 to 1) using both the minimal and π active spaces. In both molecules, the one-particle density matrices are essentially the same for singlet and triplet, and as a result, the main difference is the two-particle density matrix. As the CASSCF energy is linear in the two-particle density matrix, the CASSCF energies form a straight line along this interpolation. Interestingly, despite the nonlinear formula of the MC-PDFT functionals, the complex translation also leads to an almost perfect straight line, and for the O_2_ case, this straight line actually leads to an improved singlet–triplet gap compared to CASSCF. On the other hand, about halfway in the interpolation, when we expect Δ to pass 0, the real-only translation starts to plateau, mirroring perfectly the functional behavior in Figure 1. Because of this, the real-only translation underestimates the singlet–triplet gap, almost like an uncorrected broken symmetry DFT result would.

As we increase the active space by adding the rest of the π orbitals of O_2_, we are adding more dynamical correlation, which increases Δ. As a result, the split between real-only and complex translation narrows significantly as can be seen on the right side of the figure, and both translations give fairly similar results.

The situation is somewhat different for C_4_H_3_–NH_2_, which shows an open-shell singlet ground state, leading to a negative singlet–triplet gap. Here, CASSCF still predicts the triplet to be the ground state for the minimal active space, and while MC-PDFT reduces the gap, the correction is not enough to make the singlet the ground state. When we use the extended π active space, CASSCF correctly predicts the singlet ground state, while MC-PDFT still incorrectly predicts the triplet as ground state. In this case, the complex translation is actually slightly worse than the real-only one. Note, however, that these systems are notoriously difficult to predict correctly using DFT, being very sensitive to relaxation,^29^ which we do obviously not have with our wave function where orbitals are not optimized for MC-PDFT. Additionally, we would argue that even for these cases, the imaginary component brings a qualitatively more realistic physics for the calculations, as the linear behavior demonstrates.

The numerical results on the entire set presented in Table 3 follow similar trends. The table shows the singlet–triplet splitting errors in kcal mol^–1^ with respect to the calculated reference values found in ref (26) and the experimental value reported in ref (27). The most important observation is how the inclusion of the imaginary component in the functional translation affects every result, revealing how sensitive these spin gaps are to the way negative Δ cases are handled. The complex translation drastically reduces the errors (up to ≈16 kcal mol^–1^ for OH^+^ and tBLYP) for all the molecules following Hund’s rule (positive ST gap) and the smallest active space, while the effect is reduced with increasing active spaces. While increasing the active space may seem like a good solution, it is not always desirable or even affordable, and for this reason, we argue that the complex translation is superior. In the cases where the singlet state is lower in energy than the triplet state (negative gaps), the translation including the imaginary component increases the error but to not more than 1.0 kcal mol^–1^.

**Table 3:** Singlet–Triplet Splitting Errors in kcal mol^–1^ against Doubly Electron-Attached Coupled-Cluster Reference^26^ or Experimental References^27^ for CAS-Minimal and CAS-π Active Spaces

errors (kcal/mol)	space	CAS	tLDA	ctLDA	tPBE	ctPBE	tBLYP	ctBLYP	ref
Hund molecules									
O_2_	minimal	7.1	–7.5	–0.6	–6.7	0.6	–6.4	1.1	22.5
	π	–4.6	–3.6	–2.9	–3.1	–2.4	–3.0	–2.3	
OH^+^	minimal	4.1	–19.5	–5.8	–17.5	–2.9	–17.7	–1.8	50.5
O	minimal	6.1	–16.8	–4.3	–15.1	–1.7	–15.2	–0.7	45.4
NH	minimal	7.0	–12.1	–1.6	–10.3	1.0	–10.6	1.7	35.9
NF	minimal	7.5	–15.2	–5.1	–13.6	–2.8	–13.8	–2.3	34.3
	π	6.4	–16.5	–7.1	–14.9	–4.8	–15.1	–4.3	
C	minimal	7.3	–8.2	0.8	–6.3	3.6	–6.9	3.8	29.1
Si	minimal	7.7	–5.5	–0.5	–3.7	2.0	–4.9	1.0	17.3
C_5_H_5_^+^	minimal	15.9	–4.6	–1.5	–4.0	–0.6	–3.9	–0.3	13.9
	π	6.0	3.5	3.7	3.5	3.7	3.3	3.6	
C_4_H_2_-(13)-2CH_2_	minimal	–8.4	–1.5	–1.2	–2.0	–1.7	–2.0	–1.6	18.5
	π	3.6	–2.4	–2.3	–2.7	–2.6	–2.8	–2.8	
MAE (Hund)	minimal	7.9	10.1	2.4	8.8	1.9	9.0	1.6	
	π	5.7	7.9	4.6	7.2	3.6	7.1	3.4	
“anti”-Hund molecules									
C_4_H_4_	minimal	10.8	6.1	6.7	6.5	7.3	6.5	7.3	–4.2
	π	–6.2	4.0	4.4	4.1	4.7	4.1	4.7	
C_4_H_3_–NH_2_	minimal	16.8	10.5	11.3	11.0	12.0	11.1	12.1	–2.7
	π	–6.1	4.6	5.1	4.7	5.3	4.7	5.3	
C_4_H_3_–CHO	minimal	15.9	27.1	27.1	26.5	26.5	26.1	26.2	–3.6
	π	–6.0	4.2	4.6	4.3	4.8	4.3	4.8	
C_4_H_2_–NH_2_–CHO	minimal	–11.9	3.9	4.0	3.4	3.5	3.0	3.1	–5.7
	π	–1.0	9.9	10.3	10.0	10.4	9.9	10.3	
MAE (“anti”-Hund)	minimal	13.9	11.9	12.3	11.9	12.3	11.7	12.2	
	π	4.4	6.2	6.7	6.3	6.8	6.3	6.8	
									
MAE	minimal	9.7	10.7	5.4	9.7	5.1	9.9	4.8	
	π	5.0	6.1	5.1	5.9	4.8	5.9	4.8	

As a side note, we note that for the larger molecules, the accuracy of MC-PDFT with the small active space is very poor, regardless of the translation scheme. In the Supporting Information, we show that the errors for these systems are drastically reduced while using converged restricted open-shell Kohn–Sham triplet orbitals instead of the converged MCSCF ones. In particular, the anti-Hund MAE drops from 12.2 to 7.2 kcal mol^–1^ with the minimal active space for ctBLYP. This suggests that the current scheme using MCSCF densities is not ideal and that it would be interesting to consider the relaxation of orbitals induced by the PDFT potential.

Overall, our set of translated PDFT functionals are on average more accurate and already reasonable with the minimal active space (especially when using the DFT orbitals), which is very desirable and contrasts significantly to the results of Stoneburner et al. where very large active spaces were often required to reach the desired results.

### 4.2 Excitation Energies

While spin gaps may be considered niche examples in organic chemistry (although a more widespread issue in metal complexes), open-shell singlets are also nearly ubiquitous in excited states, which is also a key application domain of MC-PDFT.

Table 4 shows the errors for *S*_0_ → *S*_1_ excitation energies for the chosen organic molecules referenced against the values found in both the Ghosh^17^ and Schreiber^30^ benchmarks. One can see that as for the singlet–triplet splittings, the inclusion of the imaginary component in the translation is more relevant for the excitation energies calculated with the minimal active space size, with corrections between 0.1 and 0.2 eV for most cases. This means that PDFT in general reduces the active space size dependency from CASSCF, especially when the complex translation is used.

**Table 4:** *S*_0_ → *S*_1_ Excitation Energy Errors in eV for Some Molecules in the Gosh Benchmark^17^ against the Reference Contained Therein

errors (eV)	space	CAS	tLDA	ctLDA	tPBE	ctPBE	tBLYP	ctBLYP	ref
acetone	minimal	–0.32	–0.37	–0.27	–0.35	–0.24	–0.28	–0.17	4.43
	π	–0.12	0.14	0.17	0.12	0.15	0.11	0.15	
formaldehyde	minimal	–0.44	–0.52	–0.40	–0.45	–0.32	–0.39	–0.26	4.00
	π	–0.14	–0.02	0.02	0.00	0.04	0.00	0.04	
pyrazine	minimal	1.05	0.31	0.46	0.38	0.53	0.44	0.59	3.97
	π	0.77	–0.05	–0.05	0.05	0.06	0.09	0.10	
pyridazine	minimal	1.11	–0.31	–0.16	–0.21	–0.05	–0.14	0.02	3.60
	π	0.76	–0.07	–0.06	0.06	0.06	0.10	0.11	
pyridine	minimal	0.60	0.10	0.23	0.15	0.29	0.21	0.35	4.74
	π	0.26	0.65	0.66	0.64	0.65	0.60	0.61	
pyrimidine	minimal	1.20	0.22	0.30	0.28	0.37	0.36	0.45	4.18
	π	0.71	0.14	0.14	0.21	0.22	0.25	0.26	
*s*-tetrazine	minimal	1.63	–0.46	–0.35	–0.38	–0.25	–0.29	–0.16	2.25
	π	0.83	–0.12	–0.12	0.00	0.00	0.06	0.06	
ethene^a^	minimal/π	–0.17	–0.78	–0.59	–0.98	–0.77	–1.13	–0.90	8.02
butadiene	minimal	0.35	–1.34	–1.15	–1.45	–1.24	–1.53	–1.33	6.21
	π	0.39	0.63	0.62	0.58	0.58	0.54	0.54	
benzene	minimal	2.58	1.13	1.32	1.04	1.24	0.94	1.15	4.90
	π	0.04	0.40	0.41	0.39	0.40	0.35	0.36	
naphthalene	minimal	1.97	–0.43	–0.24	–0.43	–0.22	–0.45	–0.25	4.00
	π	0.19	0.41	0.42	0.41	0.41	0.38	0.39	
furan	minimal	0.28	0.39	0.46	0.18	0.25	0.06	0.14	6.06
	π	0.59	0.64	0.64	0.61	0.61	0.55	0.55	
hexatriene	minimal	0.99	–1.66	–1.46	–1.68	–1.46	–1.73	–1.50	4.93
	π	0.61	0.65	0.64	0.62	0.62	0.60	0.59	
									
MAE	minimal	1.13	0.50	0.47	0.46	0.42	0.45	0.42	
	π	0.60	0.43	0.41	0.43	0.42	0.44	0.43	

a Minimal and π active spaces are the same.

However, when the error is positive, that is, the excitation energies are overestimated, the use of the complex translation increases the excitation energy and therefore the error. This mitigates the gains from molecules where errors are negative, resulting in a MAE that is not significantly different between real-only and complex translated functionals. More precisely, if the molecules with overall positive errors are removed, we obtain MAEs of 0.74 and 0.57 eV for tBLYP and ctBLYP, respectively, which is a gain of accuracy of 0.17 eV on average, being roughly the same for the other two pairs of real-only and complex translated functionals.

As we did for the spin gap, we can investigate this issue by using KS-DFT orbitals. Specifically, looking, for example, at the first such molecule in our set, namely, pyrazine, the ground state was computed using the singlet closed-shell DFT orbitals, while the excited state was done using the triplet restricted open-shell DFT orbitals. The densities for MC-PDFT are then obtained using a simple CAS-CI. In this case, instead of an increased error of 0.15 eV, the error is instead reduced from −0.50 to −0.34 eV going from tBLYP to ctBLYP. This is consistent with the gain in accuracy between 0.1 and 0.2 eV when we move from real-only to complex translated functionals. Therefore, we argue that the poorer performance of our new set of translated functionals in these cases is due to the lack of orbital relaxation promoted by DFT, which is not present in the current MC-PDFT implementations. In a future publication, we will address this issue. For now, the main conclusion is that the better results of the real-only translation here are actually due to error cancellation with the lack of relaxation, and when done properly, the complex translation is consistently the better option and leads to a reduced active space dependence.

## 5 Conclusions

In this work, we investigated the mathematical and physical motivation to extend the translation of SDFT into PDFT functionals including the imaginary component of the spin densities when Δ < 0. This brings a more accurate physical description of open-shell singlet systems while additionally decreasing the active space size dependency. The complex translation improved singlet–triplet splitting energies up to around 16 kcal mol^–1^ and singlet–singlet excitation energies around 0.1–0.2 eV in most cases. While it may be desirable in the long run to design directly pair-density functionals, we showed that in general, a complete translation of existing Kohn–Sham functionals still satisfactorily reproduces the correct qualitative behavior. Our results also highlight the need to introduce wave function relaxation in the PDFT potential, which we will address in a future publication.

## Supporting Information Available

More detailed information on using complex number arithmetic to translate SLDA and PBE functionals into their PDFT counterparts and the importance of orbital relaxation in MC-PDFT by using the orbitals from a Kohn–Sham DFT triplet calculation (PDF)
