# Supplementary material for: Multiconfigurational Pair-Density Functional Theory Is More Complex than You May Think
Source: J Phys Chem A. 2023 Oct 27;127(44):9381–8. doi: 10.1021/acs.jpca.3c05663 (PMC10641845; doi:10.1021/acs.jpca.3c05663)
Supplement: Supplementary file 1 — jp3c05663_si_001.pdf [file jp3c05663_si_001.pdf]

Multiconfigurational Pair-Density Functional Theory is More Complex Than You May Think - Supporting Information

Gabriel L. S. Rodrigues,† Mikael Scott,† and Mickael G. Delcey*,‡

†*Division of Theoretical Chemistry and Biology, School of Engineering Sciences in Chemistry, Biotechnology and Health, KTH Royal Institute of Technology, SE-100 44 Stockholm, Sweden*

‡*Division of Theoretical Chemistry, Department of Chemistry, Lund University, SE-221 00 Lund, Sweden*

E-mail: mickael.delcey@compchem.lu.se

Contents

1 Complex arithmetic for translated functionals **S2**

1.1 General rules and Slater exchange S2

1.2 VWN3 and PBE correlation functionals S3

1.3 Full Translation of PBE exchange functional S4

2 Singlet-triplet gaps using DFT orbitals and CASCI **S5**

References **S7**

1 Complex arithmetic for translated functionals

1.1 General rules and Slater exchange

The trick to translate a functional to a complex formalism is to express the translated α and β densities in exponential form. As a reminder, any complex number $z = a + ib$ can be expressed in its polar form as $z = re^{i\theta}$ with:

$$r = \sqrt{a^2 + b^2}, \tag{S1}$$

and

$$\theta = \arctan \frac{b}{a}. \tag{S2}$$

In this form, it becomes easy to take power, multiplications and divisions. Applying the definition used in Eq. 5 from the main text we can proceed with the translation of densities for cases where $\Delta < 0$ and the "translated densities" necessarily have an imaginary component:

$$\rho_{\alpha/\beta} = \frac{\sqrt{\rho^2 - \Delta}}{2} \exp\left(\pm i \arctan\left(\frac{\sqrt{-\Delta}}{\rho}\right)\right), \tag{S3}$$

where we again assign the "+" and "-" signs to our α and β densities, respectively.

Let us illustrate this for the Slater exchange functional. The Slater functional exchange energy can be defined as:

$$E_{\text{Slater}} = -C_X \int \left(\rho_\alpha^{4/3} + \rho_\beta^{4/3}\right) d^3r, \tag{S4}$$

which becomes

$$E_{\text{Slater}} = -C_X \int \left(\frac{\rho + \sqrt{\Delta}}{2}\right)^{4/3} + \left(\frac{\rho - \sqrt{\Delta}}{2}\right)^{4/3} d^3r, \tag{S5}$$

by transforming ρ_a and ρ_b in terms of ρ and Π accordingly to Eq. 2 and using our definition of Δ from Eq. 5.

Starting from Eq. S3, the power $\frac{4}{3}$ affects r and becomes a multiplicative factor in the exponent. Then, since the α and β densities differ by the sign of the exponential, summing them gives a cosine:

$$E_{\text{Slater}} = -2C_X \int \frac{\left(\rho^2 - \Delta\right)^{2/3}}{2^{4/3}} \cos\left(\frac{4}{3}\arctan\left(\frac{\sqrt{-\Delta}}{\rho}\right)\right) d^3r, \tag{S6}$$

which is equivalent to the formula presented in Ref. 1.

Commonly, instead of spin densities, the functionals are expressed in terms of the spin polarization ζ and the spin-polarization dependent term $F(\zeta)$, which is often written as:

$$F(\zeta) = (1+\zeta)^x + (1-\zeta)^x. \tag{S7}$$

For the complex case this can be translated as:

$$F(\zeta) = 2\left(1+\eta^2\right)^{x/2} \cos\left(x \arctan\eta\right), \tag{S8}$$

where $\eta = \frac{\sqrt{-\Delta}}{\rho}$ and $\zeta = \pm i\eta$.

1.2 VWN3 and PBE correlation functionals

In the VWN3[2] (as well many others) correlation functional it is necessary to calculate the spin polarization factor ζ, which is dependent on ρ_α and ρ_β, and, therefore, needs translation. However, the ζ dependence is of the form

$$F(\zeta) = (1+\zeta)^{\frac{4}{3}} + (1-\zeta)^{\frac{4}{3}} \tag{S9}$$

Using Eq. S8, this $F(\zeta)$ term is simply translated as

$$F(\zeta) = 2(1+\eta^2)^{\frac{2}{3}} \cos\left(\frac{4}{3}\arctan\eta\right) \tag{S10}$$

The correlation functional in PBE[3] contains one such term as well as another of a similar form:

$$u(\alpha,\beta) = \frac{1}{2}\left((1+\zeta)^{\frac{2}{3}} + (1-\zeta)^{\frac{2}{3}}\right) \tag{S11}$$

which similarly translates to

$$u(\alpha,\beta) = (1+\eta^2)^{\frac{1}{3}} \cos\left(\frac{2}{3}\arctan\eta\right) \tag{S12}$$

1.3 Full Translation of PBE exchange functional

The exchange of PBE is a bit more complicated. First, following Li Manni et al, we translate the gradient of the spin-density χ_α as

$$\chi_\alpha = \frac{1}{2}\chi(1+\zeta) \tag{S13}$$

which corresponds to neglecting the explicit dependence on the gradient of ζ. The gradient is used in a dimensionless constant s_α^2 which is defined (except for a constant) as:

$$\chi_\alpha^2 \rho_\alpha^{-\frac{8}{3}} = \left(\frac{1}{2}\right)^{-\frac{2}{3}} \chi^2 \rho^{-\frac{8}{3}} (1+\zeta)^{-\frac{2}{3}} \tag{S14}$$

and for complex cases we have

$$(1+\zeta)^{-\frac{2}{3}} = (1+\eta^2)^{-\frac{1}{3}}(\cos\theta + i\sin\theta). \tag{S15}$$

with $\theta = -\frac{2}{3}\arctan\eta$ and the complex conjugate for β^2.

Being in the $a+ib$ form allows us to do additions and substractions easily, which we need

to compute $1 + \frac{\mu s^2}{\kappa}$ in the PBE exchange expression, and then we can for example convert back to exponential form to take the division in $\frac{\kappa}{1+\mu s^2/\kappa}$. This type of back-and-forth allows to extend any functional to cases where $\Delta < 0$

2 Singlet-triplet gaps using DFT orbitals and CASCI

Instead of using MCSCF orbitals and densities, we can investigate the importance of orbital relaxation in MC-PDFT by using the orbitals from a Kohn-Sham DFT triplet calculation. More specifically, in this section, we started with a triplet restricted open-shell DFT calculations as these orbitals should also be the best orbitals for the triplet MC-PDFT with the corresponding functional. We used the same orbitals for the singlet, as for a true open-shell singlet, the triplet orbitals should be also good approximations of the optimal singlet state orbitals. We then obtained the one and two-particle density matrices from a CASCI calculation for both singlet and triplet cases. The results are shown in table S1.

Table S1: Singlet-triplet splitting errors in kcal mol^{-1} against doubly electron-attached coupled-cluster reference[4] or experimental reference[5] (O_2 only), for minimal and π active spaces when using KS orbitals in a CASCI calculation.

Errors (kcal/mol)	Space	CAS	tLDA	ctLDA	tPBE	ctPBE	tBLYP	ctBLYP	Ref.
				Hund molecules					
O_2	minimal	7.1	-7.6	-0.6	-6.8	0.5	-6.5	1.2	22.5
	π	-4.6	-3.6	-3.0	-3.0	-2.5	-2.8	-2.3	
OH^+	minimal	4.1	-19.4	-5.5	-17.3	-2.6	-17.4	-1.2	50.5
O	minimal	6.1	-17.0	-4.3	-15.1	-1.7	-15.1	-0.4	45.4
NH	minimal	7.0	-12.1	-1.4	-10.3	1.2	-10.4	2.1	35.9
NF	minimal	7.5	-17.1	-9.2	-16.4	-8.2	-16.1	-7.3	34.3
	π	6.4	-15.9	-6.3	-14.6	-4.4	-14.5	-3.7	
C	minimal	7.3	-8.1	1.3	-6.2	3.9	-6.7	4.4	29.1
Si	minimal	7.7	-5.6	-0.4	-3.8	2.1	-4.8	1.3	17.3
$C_5H_5^+$	minimal	15.9	-6.8	-3.5	-6.1	-2.5	-6.0	-2.2	13.9
	π	6.0	-0.6	-0.1	-0.2	0.4	-0.1	0.5	
C_4H_2-(13)-$2CH_2$	minimal	-8.4	-10.2	-8.5	-9.6	-7.8	-9.5	-7.5	18.5
	π	3.6	0.8	0.8	0.3	0.4	0.1	0.2	
MAE (Hund)	minimal	7.9	11.5	3.8	10.2	3.4	10.3	3.1	
	π	5.7	6.7	3.1	5.9	2.4	5.8	2.2	
				"anti"-Hund molecules					
C_4H_4	minimal	10.8	6.7	7.3	7.0	7.7	7.1	7.8	-4.2
	π	-6.2	5.1	5.6	5.2	5.8	5.2	5.8	
$C_4H_3-NH_2$	minimal	16.8	5.7	6.3	6.1	6.8	6.1	6.8	-2.7
	π	-6.1	5.6	6.3	8.1	8.8	2.2	3.1	
C_4H_3-CHO	minimal	15.9	5.6	6.0	5.9	6.4	5.9	6.5	-3.6
	π	-6.0	5.7	6.1	5.9	6.4	5.7	6.2	
$C_4H_2-NH_2-CHO$	minimal	-11.9	6.7	7.4	6.8	7.7	7.0	8.0	-5.7
	π	-1.0	11.3	11.7	11.3	11.7	11.4	11.9	
MAE ("anti"-Hund)	minimal	13.9	6.1	6.7	6.4	7.2	6.5	7.2	
	π	4.4	7.5	8.0	8.4	9.0	6.4	7.0	
MAE	minimal	9.7	9.9	4.7	9.0	4.5	9.1	4.3	
	π	5.0	6.1	5.0	6.1	5.0	5.3	4.2	

References

(1) Becke, A. D.; Savin, A.; Stoll, H. Extension of the local-spin-density exchange-correlation approximation to multiplet states. *Theoretica chimica acta* **1995**, *91*, 147–156.

(2) N, L. W. A.; Nusair, D. M. Accurate spin-dependent electron liquid correlation energies for local spin density calculations: a critical analysis1. *J. Phys* **1980**, *58*, 1200.

(3) Perdew, J. P.; Burke, K.; Ernzerhof, M. Generalized Gradient Approximation Made Simple. *Phys. Rev. Lett.* **1996**, *77*, 3865–3868.

(4) Stoneburner, S. J.; Shen, J.; Ajala, A. O.; Piecuch, P.; Truhlar, D. G.; Gagliardi, L. Systematic design of active spaces for multi-reference calculations of singlet–triplet gaps of organic diradicals, with benchmarks against doubly electron-attached coupled-cluster data. *The Journal of Chemical Physics* **2017**, *147*, 164120.

(5) Bao, J. L.; Sand, A.; Gagliardi, L.; Truhlar, D. G. Correlated-Participating-Orbitals Pair-Density Functional Method and Application to Multiplet Energy Splittings of Main-Group Divalent Radicals. *Journal of Chemical Theory and Computation* **2016**, *12*, 4274–4283.
